# Rubinstein–Taybi Syndrome: A Comprehensive Analysis of a Polish Cohort with Most Cases Due to Novel *CREBBP* and *EP300* Variants

**DOI:** 10.3390/genes16101206

**Published:** 2025-10-14

**Authors:** Agata Cieślikowska, Agnieszka Madej-Pilarczyk, Piotr Iwanowski, Katarzyna Iwanicka-Pronicka, Dorota Wicher, Maria Jędrzejowska, Dorota Jurkiewicz, Marzena Gawlik, Dorota Piekutowska-Abramczuk, Paulina Halat-Wolska, Jagoda Błaszkiewicz, Izabela Mendrek, Krystyna Chrzanowska, Marlena Młynek, Piotr Stawiński, Joanna Kosińska, Małgorzata Krajewska-Walasek, Elżbieta Ciara

**Affiliations:** 1 Department of Medical Genetics, The Children’s Memorial Health Institute, Al. Dzieci Polskich 20, 04-730 Warsaw, Poland; a.cieslikowska@ipczd.pl (A.C.); a.madej-pilarczyk@ipczd.pl (A.M.-P.); p.iwanowski@ipczd.pl (P.I.); k.iwanicka-pronicka@ipczd.pl (K.I.-P.); d.wicher@ipczd.pl (D.W.); m.jedrzejowska@ipczd.pl (M.J.); m.gawlik@ipczd.pl (M.G.); d.abramczuk@ipczd.pl (D.P.-A.); p.halat@ipczd.pl (P.H.-W.); j.blaszkiewicz@ipczd.pl (J.B.); i.mendrek@ipczd.pl (I.M.); k.chrzanowska@ipczd.pl (K.C.); m.mlynek@ipczd.pl (M.M.); malgorzata.krajewskawalasek@gmail.com (M.K.-W.); 2 Department of Medical Genetics, Institute of Mother and Child, ul. Kasprzaka 17a, 01-211 Warsaw, Poland; 3 Department of Medical Genetics, Medical University of Warsaw, ul. Żwirki i Wigury 61, 02-091 Warsaw, Poland; p.stawinski@ipczd.pl (P.S.); joanna.kosinska@wum.edu.pl (J.K.)

**Keywords:** Rubinstein-Taybi syndrome, *CREBBP*, *EP300*, novel variants, next-generation sequencing, MLPA, aCGH, molecular diagnosis, population genetics

## Abstract

**Background:** Rubinstein–Taybi syndrome (RSTS) is characterized by intellectual disability, short stature, distinctive facial dysmorphism, broad thumbs/halluces, hearing loss, congenital heart or renal defects, and cryptorchidism in males. Pathogenic variants in *CREBBP* (~90% of cases) or *EP300* (~10%) underlie the disorder, with ~88% single nucleotide variants (SNVs) and ~12% copy number variants (CNVs) in *CREBBP*. **Materials and Methods:** We investigated 17 patients clinically diagnosed with RSTS at a tertiary hospital in Poland. Genetic confirmation was achieved by next-generation sequencing, multiplex ligation-dependent probe amplification (MLPA), array comparative genomic hybridization (aCGH), or Sanger sequencing. **Results:** Pathogenic variants were identified in *CREBBP* (13/17, 76%) and *EP300* (4/17, 24%). Variant types included frameshift indels (6/17, 35%), missense (4/17, 24%), nonsense (3/17, 18%), splice-site (2/17, 12%), and gross deletions (2/17, 12%). Notably, 13/17 (76%) were novel: ten in *CREBBP* (c.−49_12del, c.289C>T, c.1093_1096del, c.1094A>G, c.3178A>T, c.3401A>T, c.(3836+1_3837−1)_(4394+1_4395−1)del, c.4133+2T>G, c.4963dup, c.5028_5029dup) and three in *EP300* (c.1942C>T, c.3044_3045del, c.4713_4722del). Among the novel *CREBBP* variants, eight occurred de novo and two had unknown inheritance. Two novel *EP300* variants occurred de novo and one was of unknown origin. **Conclusions:** This first Polish RSTS cohort demonstrates a considerable proportion of gross deletions (12% overall; 15% in *CREBBP*) and an unexpectedly high rate of novel variants (76%), suggesting possible population-specific differences. These findings underscore the genetic heterogeneity of RSTS and highlight the importance of comprehensive molecular diagnostics and studies in underrepresented populations.

## 1. Introduction

Rubinstein–Taybi syndrome (RSTS) is a distinctive genetic disorder first recognized over six decades ago as a clinically identifiable pattern of malformations [1]. This rare, multisystem condition presents with a constellation of clinical and morphological features that typically enable clinical diagnosis. The estimated prevalence is approximately 1 in 100,000–125,000 live births, making RSTS an uncommon but well-defined genetic entity [2].

The molecular basis of RSTS involves monoallelic pathogenic variants in one of two key genes, *CREBBP* (accounting for the majority of cases) or *EP300*, either encoding a transcriptional coactivator with histone acetyltransferase (HAT) activity: the CREB-binding protein (CBP) and E1A binding protein p300 (EP300), respectively. Both proteins play fundamental roles in chromatin remodeling and transcriptional regulation through histone acetylation [3]. They are composed of multiple functional domains. The KIX domain mediates binding to phosphorylated CREB and other transcription factors while the bromodomain recognizes acetylated lysine residues in histones. The HAT domain catalyzes acetylation of histones and certain non-histone proteins, thereby regulating transcription. Cysteine-rich domains, including TAZ1/CH1 and TAZ2/CH3, are involved in interactions with various regulatory proteins, such as transcription factors and viral proteins. The KAT11 domain serves as the main catalytic region for acetyltransferase activity, and the IQ domain, rich in IQ motifs, participates in calmodulin binding. The NCBD/IBiD domain interacts with other transcriptional cofactors, while the Q-rich (glutamine-rich) domain functions as a transactivation region. Together, these domains form a scaffold that integrates signals from multiple transcriptional pathways, making CBP and EP300 essential for the regulation of gene expression, cell proliferation, differentiation, and DNA repair [4].

The clinical presentation of RSTS typically includes moderate to severe intellectual disability and severe expressive speech delay, short stature, and distinctive craniofacial features such as microcephaly, large anterior fontanel with delayed closure, frontal bossing, low anterior hairline, hypoplastic maxilla, and micro- or retrognathia. Patients with RSTS smile with near closure of eyes, resulting in a hallmark grimacing appearance. Ears are typically low-set and, in some cases, dysplastic. Hearing loss and recurrent otitis media are common. Ocular region abnormalities include high-arched eyebrows, long eyelashes, ptosis, epicanthal folds, and downslanting palpebral fissures, as well as ophthalmologic defects including nasolacrimal duct obstruction, strabismus, cataract, glaucoma, and coloboma. Nasal region characteristics include a beaked nose with a low-hanging columella, sometimes accompanied by a deviated nasal septum. The mouth is small, with a narrow and high-arched palate. Dental findings include crowding, talon cusps, crossbite, screwdriver-shaped permanent incisors, enamel hypoplasia, and enamel discoloration. While postnatal growth restriction is common, intrauterine growth restriction may also be observed. Hallmark skeletal findings include broad and often angulated thumbs and big toes. Behavioral and psychiatric manifestations include generally good social interactions, a short attention span, and labile mood. Motoric coordination is poor and hypotonia with hyperreflexia may be present. Seizures with EEG abnormalities can occur. A significant proportion of patients present with congenital heart defects, genitourinary anomalies (including cryptorchidism) and feeding difficulties during infancy. The multisystem nature of the disorder requires ongoing, interdisciplinary care throughout the lifespan [5].

Variants affecting specific regions of the *CREBBP* gene (i.e., exons 30–31) have been shown to cause a clinically distinct condition known as Menke–Hennekam syndrome 1 (MKHK1). Due to the high sequence homology between *CREBBP* and *EP300*, analogous variants in *EP300* (in exons 31–32) are associated with a similar phenotype, named as MKHK2. Although both these syndromes share some features with classic RSTS, their different phenotypic characteristics justify their classification as separate diagnostic entities [6,7,8].

Existing knowledge on the genetic background of RSTS remains predominantly based on cohorts from European and North American populations. Expanding research to include genetically diverse ethnic groups is essential, as population-specific variants may contribute to the broader mutational landscape of the syndrome. For example, studies from Asian populations, including Korean cohorts, have reported high rates of novel variants, with approximately 50% of identified mutations being previously unreported [9]. The continued discovery of novel pathogenic variants in RSTS is driven by expanding availability of advanced molecular diagnostics. Modern testing strategies integrate traditional sequencing with complementary methods capable of detecting larger genomic rearrangements, demonstrating that both single nucleotide variants (SNVs) and structural or copy number variants (CNVs) can be disease-causing. Comprehensive characterization of the variant spectrum across diverse populations remains essential to improving diagnostic accuracy and informing genetic counseling.

## 2. Materials and Methods

### 2.1. Patients

This study comprised 17 individuals with Rubinstein–Taybi syndrome (RSTS), all of whom were referred for clinical evaluation and molecular genetic testing at the Children’s Memorial Health Institute (CMHI) in Warsaw, Poland, between 2000 and 2025. Patients were consecutively collected over this 20-year period, reflecting both the rarity of the disorder and the progressive evolution of genetic testing technologies during this time. The study population represented a range of clinical contexts: patients with a prior clinical diagnosis of RSTS undergoing molecular confirmation, individuals with nonspecific neurodevelopmental phenotypes evaluated through reverse phenotyping, and cases in which molecular findings ultimately guided the recognition of the syndrome.

### 2.2. Genetic Analysis

Molecular diagnostics for all 17 individuals was performed using genomic DNA samples automatically extracted from the peripheral blood leukocytes with a MagNA Pure LC 2.0 (Roche Diagnostics, Risch-Rotkreuz, Switzerland) or a MagCore Nucleic Acid Extractor HF16Plus (RBC Bioscience, New Taipei City, Taiwan), according to the manufacturer’s protocol. The DNA samples were quantified using the Qubit dsDNA HS Assay Kit (Life Technologies, Eugene, OR, USA), and DNA degradation was assessed by 1% agarose gel electrophoresis. About 50 ng of high-quality genomic DNA was used for next-generation sequencing (NGS). NGS was performed with the original CMHI NGS panel of >1000 clinically relevant genes (Roche Diagnostics, Risch-Rotkreuz, Switzerland, and Twist Bioscience, San Francisco, CA, USA) (Supplementary Table S1) or a TruSight One Sequencing Panel (Illumina, San Diego, CA, USA), both encompassing *CREBBP* and *EP300* genes. Enriched libraries were paired-end-sequenced (2 × 100 bp) on the HiSeq 1500/NovaSeq 6000 (Illumina, San Diego, CA, USA), according to the manufacturer’s protocol. The average read depth was 130, with >95% of the target regions covered at a depth of 20-fold.

Raw FASTQ reads were mapped to the human genome assembly GRCh38/hg38. Variant calling was performed using multiple open-access algorithms: GATK HaplotypeCaller, MuTect2, FreeBayes, and DeepVariant to improve sensitivity for SNV and small indel detection. CNVs were analyzed using CNVkit and Decon. Alignments were visualized with the Integrative Genomics Viewer v2.16.2 [10]. The following quality control parameters were reported to evaluate the performance of the variant calling assessment: sensitivity 99.5%, precision > 99%, and F-Score > 99%. These reflect the experimentally validated results from our analysis pipeline.

Variant consequence annotation was performed using the Ensembl Variant Effect Predictor (VEP), and the changes were further annotated with multiple data repositories, including (1) frequency databases, such as the Genome Aggregation Database (gnomAD v4.1.0) and an in-house database specific to the Polish population, comprising data from >12,000 individuals suspected of having a rare genetic disease (POLdb); (2) predicted impact on protein structure and function, assessed with in silico tools, including machine learning meta-scores (BayesDel, REVEL) and individual predictors (AlphaMissense, CADD, EIGEN, FATHMM-MKL, MutationTaster, PolyPhen-2, SIFT); variants occurring at splice sites, analyzed using SpliceAI, ADA, MaxEntScan, Pangolin, and RF to assess their potential impact on normal splicing; (3) occurrence in different reference databases, such as ClinVar, the Leiden Open Variation Database (LOVD), and the Human Gene Mutation Database (HGMD) [11].

Finally, variants were interpreted based on the guidelines of the American College of Medical Genetics and Genomics and the Association for Molecular Pathology (ACMG/AMP), as well as the Association for Clinical Genomic Science (ACGS) [12,13]. According to these criteria, benign and likely benign variants were filtered out, leaving only pathogenic (P), likely pathogenic (LP) changes, and variants of uncertain significance (VUS). Variants considered (potentially) disease-causing (P/LP) were correlated with the phenotype and validated in probands and segregated in family members using bi-directional Sanger sequencing with the BigDye Terminator v3.1 Kit (Applied Biosystems, Waltham, MA, USA) on the ABI 3130 Genetic Analyzer (Applied Biosystems, Waltham, MA, USA), according to the manufacturer’s protocol.

Large-scale genomic alterations were assessed through multiplex ligation-dependent probe amplification (MLPA) and array comparative genomic hybridization (aCGH) to identify deletions and duplications in *CREBBP* and *EP300* genes [14]. These methodologies were particularly crucial given that CNVs represent a substantial proportion of genetic background in RSTS patients and may escape detection through standard sequencing approaches.

MLPA reaction was performed using the SALSA MLPA P096, P313 and P333 Kits (MRC Holland, Amsterdam, The Netherlands). Denaturation, hybridization to probes, ligation and amplification were performed according to the manufacturer’s recommendations. DNA samples with known aberration were included as a control in each reaction’s run. The GeneMarker v2.2.0 software (Soft Genetics, LLC, State College, PA, USA) was used for data analysis. Peak ratio 0.7–1.3 for loss and gain detection were used as thresholds.

MLPA was performed in six patients (P03, P06, P07, P08, P09, P14) who had been analyzed with the TruSight One Sequencing Panel, due to its lower sensitivity for CNV detection compared with the CMHI 1000-gene NGS panel. In addition, in patients P01, P02, P04, and P05, analyzed in the early 2000s before the wide availability of NGS, the CREBBP gene was examined sequentially by MLPA and by Sanger sequencing of all exons and exon–intron boundaries.

The whole genome aCGH procedure was performed following the manufacturer’s instructions (SurePrint G3 ISCA V2 CGH Microarray Kit; Agilent Technologies, Santa Clara, CA, USA). The 8 × 60K slides were scanned on a NimbleGen 200 Microarray Scanner (Roche Nimblegen, Madison, WI, USA). Feature extraction and data analysis were carried out with Agilent CytoGenomics 5.2.1.4 software (Agilent Technologies, Santa Clara, CA, USA) using default analysis settings. The array CGH results were analyzed with the UCSC hg19 assembly.

Parental samples were analyzed when available by targeted Sanger sequencing of the respective *CREBBP/EP300* exon or MLPA/aCGH to establish inheritance patterns and confirm de novo status. Cases lacking parental samples were classified as having unknown inheritance patterns.

The nomenclature of reported molecular variants follows the Human Genome Variation Society (HGVS) guidelines using the MANE Select human *CREBBP* and *EP300* reference sequence, respectively: NM_004380.3 and NM_001429.4 (for cDNA) and NP_004371.2 and NP_001420.2 (for protein).

## 3. Results

### 3.1. Molecular Findings

The *CREBBP* and *EP300* pathogenic variants identified in our cohort of 17 clinically diagnosed RSTS patients are summarized in Table 1 and Supplementary Tables S2 and S3. Pathogenic or likely pathogenic variants were detected in *CREBBP* in 13 patients (76%) and in *EP300* in 4 patients (24%). Across both genes, variant types included frameshift indels in 6/17 patients (35%), missense variants in 4/17 (24%), nonsense variants in 3/17 (18%), splice-site variants (including one start-loss variant) in 2/17 (12%), and gross deletions in 2/17 (12%), while all CNVs occurred in *CREBBP* (2/13; 15%).

In *CREBBP*, the SNVs spectrum comprised two nonsense variants, four frameshift variants, three missense variants, one splice-site variant, one start-loss, and two gross deletions. The two CNVs involved multi-exons (5–31 and 22–26, respectively) losses within the 16p13.3 region of *CREBBP*. Among the 13 *CREBBP* cases (including both novel and previously reported variants), 10 variants were confirmed de novo, while in 3 cases, inheritance could not be determined due to missing biparental DNA.

In *EP300*, the spectrum comprised two frameshift deletions, one nonsense, and one missense variant. Two of the three novel *EP300* variants occurred de novo. For the single frameshift variant (P16), parental samples were unavailable, leaving its inheritance status undetermined.

Overall, 13 of the 17 identified variants (76%) were novel, substantially broadening the known pathogenic variants landscape of RSTS. These included 10 previously unreported changes in *CREBBP* (c.−49_12del, c.289C>T, c.1093_1096del, c.1094A>G, c.3178A>T, c.3401A>T, c.(3836+1_3837−1)_(4394+1_4395−1)del, c.4133+2T>G, c.4963dup, c.5028_5029dup) and 3 in *EP300* (c.1942C>T, c.3044_3045del, c.4713_4722del). The proportion of gross deletions (15%) was consistent with frequencies previously reported for *CREBBP*-associated RSTS [5,15,16].

Among the known variants, a gross deletion of *CREBBP* (c.(1216+1_1217–1)_*1del; exons 5–31) has been previously described in patients with RSTS [17]. The frameshift variant c.5837dup (p.Pro1947Thrfs*19) has also been reported [18,19,20,21] and submitted to ClinVar. The missense variants c.3832G>A (p.Glu1278Lys) and c.3205G>A (p.Asp1069Asn) are listed in ClinVar [22,23].

**Figure 1:**
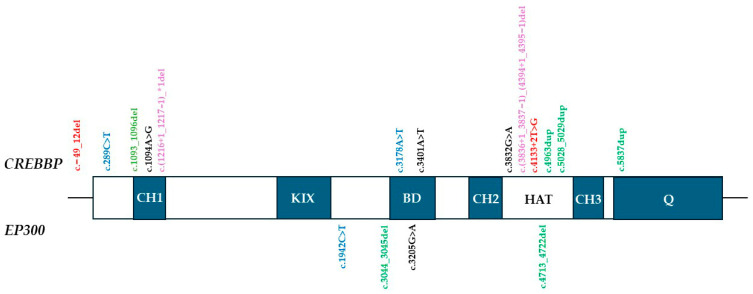
Domain distribution of the CREBBP and EP300 variants. Abbreviations: HAT: histone acetyltransferase domain (CBP/EP300); BD: bromodomain; KIX: CREB-binding domain; CH: cysteine-histidine-rich domain; Q: glutamine rich domain, containing NCBD/IBiD domain (nuclear receptor coactivator binding/interferon response factor binding domain); Variants: red—splice-site; blue—nonsense; green—frameshift del/dup; black—missense; violet—gross deletion. * denotes a stop (termination) codon according to HGVS nomenclature.

The distribution of identified variants across functional domains of CBP and EP300 is illustrated in Figure 1. Truncating variants were scattered throughout both proteins, consistent with global loss-of-function. In contrast, the two novel missense variants showed domain-specific localization: one (c.3401A>T; p.Asp1134Val) within the bromo-domain, which mediates recognition of acetylated histone lysines, and the other (c.1094A>G; p.His365Arg) in the CH1 domain (also known as TAZ1), a cysteine-rich region facilitating protein–protein interactions with transcription factors. Both residues are highly conserved across species [24,25]. Although most reported pathogenic missense variants in RSTS cluster within the HAT/KAT domain, our findings support the notion that changes in other domains, including CH1, may also contribute to disease pathogenesis [26]. In contrast, the remaining truncating variants (nonsense, frameshift, splice-site, and multi-exon deletions) represent clear loss-of-function alleles and were therefore not further discussed in detail.

The detailed molecular findings for all patients are presented in Supplementary Table S3, while the corresponding clinical characteristics are described below.

**Table 1:** Genetic variants in CREBBP and EP300 and associated clinical features in patients with Rubinstein–Taybi syndrome.

Patient ID	Gender	Genotype (cDNA)	Protein Effect	Typical Facial Dysmorphism #	Short Stature	Microcephaly	Broad Thumbs/Halluces	Cardiac Anomalies	Skeletal and/or Dental Anomalies	Hypotonia	NDD/ID	Hypertrichosis
CREBBP NM_004380.3, NP_004371.2, chr16(GRCh38):g.3725054–3880713												
P01	F	c.[4963dup];[=]	p.(Leu1655Profs*5)	Y	Y	Y	Y/Y	Y	Y	Y	Y	Y
P02	M	c.[3178A>T];[=]	p.(Lys1060*)	Y	Y	Y	Y/Y	Y	N	Y	Y	Y
P03	F	c.[5028_5029dup];[=]	p.(Glu1677Glyfs*68)	Y	Y	Y	Y/Y	Y	Y	Y	Y	Y
P04	M	c.[(1216+1_1217−1)_*1del];[=]	p.?	Y	Y	Y	Y/Y	Y	N	N	Y	Y
P05	M	c.[(3836+1_3837−1)_(4394+1_4395−1)del];[=]	p.?	Y	Y	Y	Y/Y	Y	Y	Y	Y	Y
P06	M	c.[4133+2T>G];[=]	p.?	Y	Y	Y	Y/Y	Y	Y	Y	Y	Y
P07	M	c.[289C>T];[=]	p.(Gln97)*	Y	Y	Y	Y/Y	N	Y	Y	Y	Y
P08	M	c.[−49_12del];[=]	p.? start loss	Y	Y	Y	N/Y	N	Y	Y	Y	Y
P09	M	c.[1093_1096del];[=]	p.(His365Serfs*23)	Y	Y	Y	N/N	N	Y	Y	Y	N
P10	F	c.[5837dup];[=]	p.(Pro1947Thrfs*19)	Y	Y	Y	Y/Y	Y	NA	Y	Y	Y
P11	F	c.[3832G>A];[=]	p.(Glu1278Lys)	Y	Y	Y	Y/Y	Y	Y	Y	Y	Y
P12	F	c.[3401A>T];[=]	p.(Asp1134Val)	Y	Y	N	Y/Y	N	N	N	Y	Y
P13	F	c.[1094A>G];[=]	p.(His365Arg)	N	Y	Y	N/Y	N	N	Y	Y	N
EP300 NM_001429.4, NP_001420.2, chr22(GRCh38):g41092510–41180077												
P14	F	c.[1942C>T];[=]	p.(Arg648*)	Y	Y	Y	N/N	N	Y	Y	Y	N
P15	F	c.[4713_4722del];[=]	p.(Gly1572Metfs*23)	Y	Y	Y	N/Y	N	Y	Y	Y	Y
P16	F	c.[3044_3045del];[=]	p.(Arg1015Lysfs*3)	Y	N	Y	Y/Y	Y	N	N	Y	N
P17	F	c.[3205G>A];[=]	p.(Asp1069Asn)	N	N	N	N/Y	Y	N	Y	Y	N

Abbreviations: Y: present; N: absent; N: not applicable; NDD: neurodevelopmental delay; ID: intellectual disability; M: male; F: female. # Typical facial dysmorphism according to Lacombe et al. [26]: highly arched eyebrows; downslanted palpebral fissures; convex nasal ridge; columella below alae nasi; highly arched palate; typical smile. * denotes a stop (termination) codon according to HGVS nomenclature

### 3.2. Clinical Characteristics

Clinical characteristics of our cohort with RSTS are summarized in Table 1, complementing the molecular findings. Percentages are calculated based on the number of patients for whom relevant clinical information was available. Additional phenotypic details for each patient, not included in the main summary table, are provided in Supplementary Table S2.

All individuals exhibited a phenotype consistent with the established diagnostic criteria for RSTS, with variable expressivity across the analyzed group. Evaluation of facial dysmorphism was based on the cardinal craniofacial features defined by Lacombe et al. [25], namely highly arched eyebrows, downslanted palpebral fissures, convex nasal ridge, columella below alae nasi, highly arched palate, and a characteristic smile. Typical facial dysmorphism was observed in 15/17 patients (88.2%) and was frequently accompanied by additional craniofacial findings such as low anterior hairline, thin upper lip, small mouth, long philtrum, long eyelashes, thick eyebrows, dysplastic ears, flat facial capillary malformations, and epicanthal folds. Micrognathia or retrognathia was identified in 11/17 patients (64.7%), representing a relatively common craniofacial feature within our cohort.

Short stature was observed in 15/17 patients (88.2%), and microcephaly in 15/17 (88.2%). Postnatal growth restriction was particularly frequent, affecting 16/17 patients (94.1%), whereas prenatal growth restriction occurred in 9/17 (52.9%). Broad thumbs and/or halluces, one of the cardinal features of RSTS, were present in 15/17 patients (88.2%), typically readily apparent on clinical examination and often accompanied by angular deviation, brachydactyly, clinodactyly of the fifth finger, or a sandal gap. Additional hand and foot anomalies included short feet, camptodactyly, pes planovalgus, preaxial polydactyly, and syndactyly.

Neurodevelopmental delay or intellectual disability was present in all patients (17/17, 100%), ranging from mild to severe. Hypotonia was reported in 14/17 patients (82.4%). Behavioral problems were common and included anxiety, aggression, impulsivity, hyperactivity, social withdrawal, stereotypies, and attention deficit. Structural brain anomalies, such as agenesis of the corpus callosum, abnormal myelination, and spina bifida, were noted in several cases. Seizures were reported in isolated patients. Additionally, one patient developed a medulloblastoma, consistent with the rarely reported tumor predisposition in RSTS [16,27].

Cardiac anomalies were documented in 10/17 patients (58.8%), including atrial septal defect (ASD), ventricular septal defect (VSD), patent ductus arteriosus (PDA), patent foramen ovale (PFO), and complex combinations thereof. None of the identified defects were of critical severity.

Genitourinary anomalies were observed exclusively in males, affecting 6/7 (85.7%), with cryptorchidism being the sole finding. No renal or other genital tract malformations were identified. Skeletal and/or dental anomalies were evaluated in 13 patients, with abnormalities identified in 10 (76.9%). The most frequent findings included dental caries, talon cusps, scoliosis, and enamel hypoplasia.

Hypertrichosis was present in 12/17 patients (70.6%) and, as one of the recognized diagnostic features of RSTS, contributed to the clinical recognition of the syndrome.

Feeding problems were reported in 12/16 patients (75.0%), most commonly involving gastroesophageal reflux, constipation, or recurrent vomiting.

Ophthalmologic abnormalities were identified in 9/13 patients (69.2%) and were heterogeneous, with the most frequent findings being strabismus and nasolacrimal duct obstruction, followed by recurrent conjunctivitis, photophobia, glaucoma, and optic nerve atrophy. Hearing impairment was documented in 4/16 patients (25%), and recurrent otitis media in 6/15 (40%).

Overall, the cohort exhibited the key diagnostic features of RSTS, alongside a variable spectrum of additional systemic manifestations.

**Figure 2:**
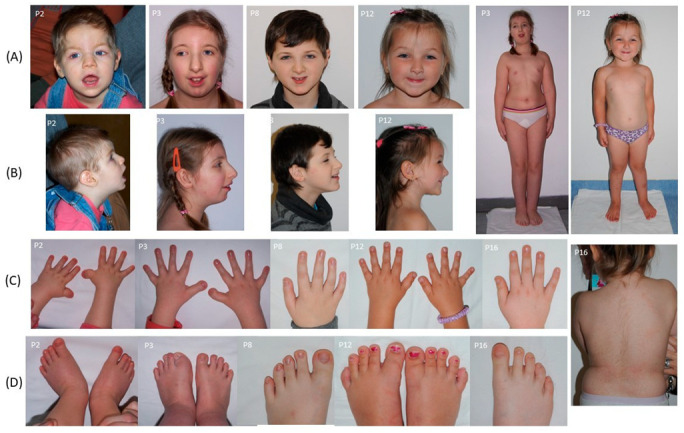
Clinical features of selected patients with Rubinstein–Taybi syndrome from the studied cohort. (**A**) Frontal views showing characteristic craniofacial dysmorphism, including arched eyebrows, downslanted palpebral fissures, and thin upper lip, in patients P2, P3, P8, and P12; full-body views of P3 and P12. (**B**) Lateral views of the same patients as in panel A. (**C**) Hands of patients P2, P3, P8, P12, and P16, showing broad thumbs and radial deviation. (**D**) Feet of patients P2, P3, P8, P12, and P16, demonstrating broad halluces; back view of P16 showing hypertrichosis.

Representative clinical photographs of the patients, illustrating the characteristic craniofacial features and limb anomalies, are presented in Figure 2.

## 4. Discussion

This study offers a detailed molecular and phenotypic characterization of RSTS in a Polish single-center cohort, adding to the limited literature on Central and Eastern European populations [28]. A striking finding was the high proportion of novel variants (76%), underscoring the potential for undiscovered pathogenic changes, particularly in populations that remain underrepresented in current genomic databases [5,16]. Our findings are consistent with prior observations from underrepresented populations, such as Korean cohorts, where a similarly high rate of novel variants was observed [9]. This underscores the value of expanding variant databases beyond Western populations to improve diagnostic accuracy and broaden our understanding of disease mechanisms.

Our cohort showed a notably higher proportion of *EP300* pathogenic variants (24%) compared to the typically reported 8–10% in the literature [16]. This discrepancy may reflect the use of more advanced detection methods, including comprehensive NGS panels and routine CNV analysis via MLPA and aCGH. Alternatively, the increased *EP300* rate could result from population-specific variation or an ascertainment bias, given that patients were recruited over an extended time period, potentially enriching for diagnostically complex cases.

We observed a balanced distribution of variant types, including frameshift, nonsense, missense, splice-site variants, and gross deletions. The proportion of gross deletions identified in our cohort was consistent with previous reports, reflecting the importance of applying MLPA and aCGH alongside NGS to reliably detect CNVs that might be missed by sequencing alone. While future widespread use of whole-genome sequencing may obviate the need for such complementary assays, our findings highlight the continued relevance of integrated diagnostic strategies in capturing the full spectrum of *CREBBP*- and *EP300*-related alterations.

In our cohort, no clear genotype–phenotype correlations were apparent. Patients with *CREBBP* or *EP300* variants of different classes (truncating, missense, splice-site variants, and gross deletions) exhibited a largely overlapping phenotypic spectrum. This is consistent with previous studies, which similarly reported that variant type, location, or deletion size did not consistently predict clinical severity or specific features in RSTS [29,30]. Such variability highlights the influence of additional genetic, epigenetic, or environmental factors on phenotypic expression in this syndrome.

Clinically, our findings reaffirm the core phenotypic features of RSTS, including short stature, microcephaly, characteristic facial dysmorphism, broad thumbs and/or halluces, and intellectual disability. Feeding difficulties, hypotonia, and cardiac anomalies were also frequent, underscoring the need for early multidisciplinary care and routine cardiac screening. Maternal preeclampsia and keloids, previously reported as occasional features of RSTS, were not observed in our cohort [5,31].

The variability of intellectual disability and the presence of behavioral features such as anxiety and aggression align with recent descriptions of neuropsychiatric manifestations in RSTS [32,33].

Overall, the phenotypic spectrum in our patients closely resembled that reported in earlier European series [2,15,16].

Long-term follow-up of several adult patients in our cohort provided additional insights into the natural history of RSTS. With increasing age, behavioral manifestations tended to become less disruptive, although autistic features and lack of expressive speech often persisted. Interestingly, some patients demonstrated preserved motor abilities, such as swimming skills, despite coexisting balance difficulties. These anecdotal observations, while not systematically assessed, are consistent with previous reports describing evolving behavioral phenotypes and functional adaptation in adults with RSTS [33].

Minor differences, such as a slightly higher frequency of cardiac anomalies and cryptorchidism in our cohort, are most likely attributable to cohort size and ascertainment rather than true population-specific effects.

Most variants in our cohort occurred de novo (71% of tested cases), consistent with previous reports and with direct implications for genetic counseling, as the recurrence risk for parents is generally low. However, in several patients, inheritance could not be determined due to the unavailability of parental samples, which remains a common limitation in retrospective genetic studies.

Several limitations should be considered. The retrospective design introduces potential selection bias, and diagnostic methods varied over the 25-year recruitment period, potentially affecting detection sensitivity. Limited availability of phenotypic data and incomplete parental testing are additional constraints.

Nonetheless, this study contributes significantly to the understanding of RSTS genetics in a previously underrepresented population. The identification of multiple novel variants provides opportunities for future functional studies and reinforces the importance of applying comprehensive genetic testing strategies that include both sequence and CNV analysis. Further large-scale, population-based studies will be necessary to explore genotype–phenotype correlations and better define regional molecular patterns in RSTS.

Overall, our data highlight the need for inclusive, population-specific genomic studies to refine diagnostic strategies and patient care in RSTS.

## 5. Conclusions

We report the spectrum of *CREBBP* and *EP300* molecular variants in a cohort of 17 Polish patients with Rubinstein–Taybi syndrome. Our findings confirm that *CREBBP* pathogenic variants are the predominant cause of RSTS, while *EP300* variants accounted for a higher proportion (24%) than typically reported. Multiple novel causative variants were identified in both *CREBBP* and *EP300*.

Novel variant discovery was particularly striking, with 13 of 17 variants (76%) being previously unreported, substantially expanding the known molecular spectrum associated with the syndrome. Comprehensive genetic testing combining NGS, MLPA/aCGH or Sanger sequencing proved highly effective in detecting pathogenic variants across a broad range of types, including frameshift, nonsense, missense, splice-site variants, and gross deletions.

The proportion of gross deletions (15%) was consistent with the range previously reported for *CREBBP*-associated RSTS (10–15%), likely reflecting the systematic inclusion of CNV analysis in all cases. The majority of variants occurred de novo, consistent with the predominantly sporadic nature of the syndrome.

Despite considerable genetic heterogeneity, clinical features in our cohort were largely consistent with previous descriptions, and no clear genotype–phenotype correlations were observed. This study contributes valuable new molecular data from a previously underrepresented population and underscores the need for more inclusive genetic research. The identification of novel pathogenic variants significantly enriches the current understanding of *CREBBP*- and *EP300*-related RSTS and highlights the ongoing need for detailed molecular characterization across diverse populations.

## Data Availability

The original contributions presented in this study are included in the article/Supplementary Materials. Further inquiries can be directed to the corresponding author.

## Supplementary Materials

The following supporting information can be downloaded at: https://www.mdpi.com/article/10.3390/genes16101206/s1, Supplementary Table S1. The original Children’s Memorial Health Institute NGS panel of >1000 clinically relevant genes; Supplementary Table S2. Comprehensive overview of *CREBBP* and *EP300* variants and clinical features of the studied RSTS cohort; Supplementary Table S3. Characteristics of molecular variants identified in a group of 17 probands with RSTS. References [10,11,12,17,18,19,20,21,22,23] are cited in the supplementary materials.

## Abbreviations

The following abbreviations are used in this manuscript:

ACGS	Association for Clinical Genomic Science
ACMG	American College of Medical Genetics and Genomics
aCGH	array comparative genomic hybridization
AMP	Association for Molecular Pathology
ASD	atrial septal defect
BD	bromodomain
CBP	CREB-binding protein
CH	cysteine-histidine-rich domain
CMHI	Children’s Memorial Health Institute
CNV	copy number variant
CREB	cyclic AMP response element-binding protein
EEG	electroencephalography
EP300	E1A binding protein p300
HAT	histone acetyltransferase
HGMD	Human Gene Mutation Database
HGVS	Human Genome Variation Society
ID	intellectual disability
KIX	CREB-binding domain
LP	likely pathogenic
LOVD	Leiden Open Variation Database
MLPA	multiplex ligation-dependent probe amplification
MKHK	Menke–Hennekam syndrome
N	absent
NA	not applicable
NCBD/IBiD	nuclear coactivator binding domain/interferon-binding domain
NDD	neurodevelopmental delay
NGS	next-generation sequencing
P	pathogenic
PDA	patent ductus arteriosus
PFO	patent foramen ovale
POLdb	Polish rare disease database
RSTS	Rubinstein–Taybi syndrome
Q	glutamine-rich domain
SNV	single nucleotide variant
TAZ	transcriptional adapter zinc-binding domain
VEP	Variant Effect Predictor
VSD	ventricular septal defect
VUS	variant of uncertain significance
Y	present
